# Light Cannabis Use and the Adolescent Brain: An 8-years Longitudinal Assessment of Mental Health, Cognition, and Reward Processing

**DOI:** 10.1007/s00213-024-06575-z

**Published:** 2024-03-26

**Authors:** Inês Macedo, Tiago O. Paiva, Rita Pasion, Laura Daedelow, Andreas Heinz, Ana Magalhães, Tobias Banaschewski, Arun L. W. Bokde, Sylvane Desrivières, Herta Flor, Antoine Grigis, Hugh Garavan, Penny Gowland, Rüdiger Brühl, Jean-Luc Martinot, Marie-Laure Paillère Martinot, Eric Artiges, Frauke Nees, Dimitri Papadopoulos Orfanos, Tomáš Paus, Luise Poustka, Sarah Hohmann, Nathalie Holz, Juliane H. Fröhner, Michael N. Smolka, Nilakshi Vaidya, Henrik Walter, Robert Whelan, Gunter Schumann, Fernando Barbosa, IMAGEN Consortium, Gareth J. Barker, Herve Lemaitre, Sabina Millenet, Lauren Robinson, Jeanne M. Winterer

**Affiliations:** 1 Laboratory of Neuropsychophysiology, Faculty of Psychology and Educational Sciences (Laboratory of Neuropsychophysiology), University of Porto, Rua Alfredo Allen, 4200-135 Porto, Portugal; 2 Addiction Biology Group, i3S-Instituto de Investigação E Inovação Em Saúde, Porto, Portugal; 3 HEI-LAB, Lusófona University, Porto, Portugal; 4 Department of Psychiatry and Psychotherapy CCM, Charité – Universitätsmedizin Berlin, corporate member of Freie Universität Berlin, Humboldt-Universität Zu Berlin, and Berlin, Institute of Health, Berlin, Germany; 5 Instituto de Biologia Molecular E Celular (IBMC), University of Porto, Porto, Portugal; 6 Instituto de Ciências Biomédicas de Abel Salazar (ICBAS), University of Porto, Porto, Portugal; 7 Department of Child and Adolescent Psychiatry and Psychotherapy, Central Institute of Mental Health, Medical Faculty Mannheim, Heidelberg University, Square J5, 68159 Mannheim, Germany; 8 Discipline of Psychiatry, School of Medicine and Trinity College Institute of Neuroscience, Trinity College Dublin, Dublin, Ireland; 9 Centre for Population Neuroscience and Precision Medicine (PONS), Institute of Psychiatry, Psychology & Neuroscience, SGDP Centre, King’s College London, London, UK; 10 Institute of Cognitive and Clinical Neuroscience, Central Institute of Mental Health, Medical Faculty Mannheim, Heidelberg University, Square J5, Mannheim, Germany; 11 Department of Psychology, School of Social Sciences, University of Mannheim, 68131 Mannheim, Germany; 12 NeuroSpin, CEA, Université Paris-Saclay, 91191 Gif-Sur-Yvette, France; 13 Departments of Psychiatry and Psychology, University of Vermont, Burlington, VT 05405 USA; 14 Sir Peter Mansfield Imaging Centre School of Physics and Astronomy, University of Nottingham, University Park, Nottingham, UK; 15 Institut National de La Santé Et de La Recherche Médicale, INSERM U 1299 Trajectoires Développementales & Psychiatrie, CNRS; EcoleNormaleSupérieure Paris-Saclay, Centre Borelli, University Paris-Saclay, Gif-Sur-Yvette, France; 16 Institut National de La Santé Et de La Recherche Médicale, INSERM U 1299 Trajectoires Développementales & Psychiatrie, University Paris-Saclay, CNRS; Ecole Normale Supérieure Paris-Saclay, Centre Borelli; Gif-Sur-Yvette, Department of Child and Adolescent Psychiatry, Pitié-Salpêtrière Hospital, and AP-HP. Sorbonne University, Paris, France; 17 Institut National de La Santé Et de La Recherche Médicale, INSERM U 1299 Trajectoires Développementales & Psychiatrie, CNRS; EcoleNormaleSupérieure Paris-Saclay, Centre Borelli; Gif-Sur-Yvette; and Psychiatry Department, EPS Barthélémy Durand, University Paris-Saclay, Etampes, France; 18 Departments of Psychiatry and Neuroscience, Faculty of Medicine and Centre Hosptalier, Universitaire Sainte-Justine, University of Montreal, Montreal, QC Canada; 19 Departments of Psychiatry and Psychology, University of Toronto, Toronto, ON Canada; 20 Department of Child and Adolescent Psychiatry and Psychotherapy, University Medical Centre Göttingen, Von-Siebold-Str. 5, 37075 Göttingen, Germany; 21 Department of Psychiatry and Neuroimaging Center, Technische Universität Dresden, Dresden, Germany; 22 Centre for Population Neuroscience and Stratified Medicine (PONS), Department of Psychiatry and Neuroscience, Charité Universitätsmedizin, Berlin, Germany; 23 School of Psychology and Global Brain Health Institute, Trinity College Dublin, Dublin, Ireland; 24 Centre for Population Neuroscience and Precision Medicine (PONS), Institute for Science and Technology of Brain-Inspired Intelligence (ISTBI), Fudan University, Shanghai, China; 25 Institute of Medical Psychology and Medical Sociology, University Medical Center Schleswig Holstein, Kiel University, Kiel, Germany; 26 Department of Child and Adolescent Psychiatry Psychotherapy and Psychosomatics, University Medical Center Hamburg-Eppendorf, Hamburg, Germany; 27 Physikalisch-Technische Bundesanstalt, Braunschweig und Berlin, Germany

**Keywords:** Cannabis, Reward Processing, Psychopathology, Cognition, Longitudinal, fMRI, Adolescents

## Abstract

**Rationale:**

For decades, cannabis has been the most widely used illicit substance in the world, particularly among youth. Research suggests that mental health problems associated with cannabis use may result from its effect on reward brain circuit, emotional processes, and cognition. However, findings are mostly derived from correlational studies and inconsistent, particularly in adolescents.

**Objectives and Methods:**

Using data from the IMAGEN study, participants (non-users, persistent users, abstinent users) were classified according to their cannabis use at 19 and 22 years-old. All participants were cannabis-naïve at baseline (14 years-old). Psychopathological symptoms, cognitive performance, and brain activity while performing a Monetary Incentive Delay task were used as predictors of substance use and to analyze group differences over time.

**Results:**

Higher scores on conduct problems and lower on peer problems at 14 years-old (*n* = 318) predicted a greater likelihood of transitioning to cannabis use within 5 years. At 19 years of age, individuals who consistently engaged in low-frequency (i.e., light) cannabis use (*n* = 57) exhibited greater conduct problems and hyperactivity/inattention symptoms compared to non-users (*n* = 52) but did not differ in emotional symptoms, cognitive functioning, or brain activity during the MID task. At 22 years, those who used cannabis at both 19 and 22 years-old *n* = 17), but not individuals that had been abstinent for ≥ 1 month (*n* = 19), reported higher conduct problems than non-users (*n* = 17).

**Conclusions:**

Impairments in reward-related brain activity and cognitive functioning do not appear to precede or succeed cannabis use (i.e., weekly, or monthly use). Cannabis-naïve adolescents with conduct problems and more socially engaged with their peers may be at a greater risk for lighter yet persistent cannabis use in the future.

**Supplementary Information:**

The online version contains supplementary material available at 10.1007/s00213-024-06575-z.

Preliminary results of the current work were presented as a poster at the 2022 European Society of Cognitive and Affective Neuroscience Conference.

Cannabis exerts its effects in humans mainly through actions from its main psychoactive compound – THC (delta-9-tetrahydrocannabinol) – on CB1 cannabinoid receptors (Prus 2018). The endocannabinoid system, namely brain areas with the densest CB1 cannabinoid binding (e.g., basal ganglia, cerebral cortex, and striatum), appears to be involved and affect several cognitive, behavioral, emotional, and physiological processes, including reward processing (Covey et al. 2015; Fernández-Ruiz et al. 2000; Herkenham et al. 1990; Meyer et al. 2018; Pertwee 1997; Prus 2018). Investigating the effects of cannabis on the brain’s reward circuit can provide insights into its potentially addictive properties, since this circuit is broadly affected by both natural and artificial reinforcers, including drugs of abuse.

Studies using functional Magnetic Resonance Imaging (fMRI) have employed the Monetary Incentive Delay (MID) task to investigate gain anticipation and feedback processing (Knutson et al. 2000). Gain anticipation is expected to increase activity in the ventral striatum (VS) – particularly the nucleus accumbens (NAc) (Balodis and Potenza 2015; Knutson et al. 2000), while feedback-reward processing (i.e., gains) seem to elicit greater activation in the prefrontal cortex (PFC) (Knutson and Greer 2008). Therefore, the MID task allows us to investigate how brain circuits operate to evaluate rewarding stimuli (Haber and Knutson 2010), providing a neural account of the subjective value of rewards and the cues predicting them. Nonetheless, chronic effects of cannabis during reward anticipation remain unclear. There are studies reporting no differences between individuals who use cannabis and controls (Enzi et al. 2015; Jager et al. 2013; Karoly et al. 2015; Nestor et al. 2020; Skumlien et al. 2022; Tong et al. 2020; Yip et al. 2014), while others report increased activity in the VS and cerebellum (Nestor et al. 2010), and others decreased activity in PFC and/or striatal areas (Spechler et al. 2020; van Hell et al. 2010). Fewer studies have investigated reward feedback in individuals who use cannabis, but findings are also inconsistent. Both increased (Skumlien et al. 2022; van Hell et al. 2010) and decreased (Nestor et al. 2010; van Hell et al. 2010; Yip et al. 2014) activity in PFC, limbic, and sensorimotor regions are reported, as well as non-significant results (Filbey and Yezhuvath 2013; Jager et al. 2013; Skumlien et al. 2022; Tong et al. 2020; Yip et al. 2014). Since these findings come from cross-sectional investigations it is still necessary to assess if group differences originate from the: (a) neurotoxic effects of cannabis, and/or (b) preexisting individual differences, namely in the reward system, psychopathological symptoms, and/or cognitive functioning (Skumlien et al. 2021).

Longitudinal studies using the MID task (Cope et al. 2019; Martz et al. 2016, 2018, 2021) have reported mixed findings during reward anticipation: decreased NAc activity over time in individuals who use cannabis (Martz et al. 2016); non-significant predictive effects or higher VS activation with greater cannabis use (Martz et al. 2018, 2021). However, these participants had already initiated cannabis use, making it impossible to distinguish between the neurotoxic effects of cannabis and preexisting individual differences in the reward system. More longitudinal studies are needed to follow participants from before to after the onset of cannabis use, which usually begins during adolescence – an important neurodevelopment period (Ellingson et al. 2021; Meyer et al. 2018; Stringfield and Torregrossa 2021). The ongoing changes in adolescents’ brain systems can lead to increased risk-taking and reward-seeking, making them more vulnerable to hazardous behaviors, such as drug misuse (Steinberg 2008). The endocannabinoid system has a crucial role in regulating brain development and can produce long-lasting functional changes in synaptic processes (Fernández-Ruiz et al. 2000; Lupica et al. 2004); thus, exogenous cannabinoids may disrupt the developmental processes (Rubino and Parolaro 2016).

The relationship between cognitive impairments—which are closely interrelated with brain functioning—and cannabis use is also subject of ongoing debate, particularly concerning its potential for predicting cannabis use initiation versus it being a consequence of cannabis consumption. Preexisting cognitive impairments may predict cannabis use onset, for example, poor executive functioning in childhood seems to be associated with cannabis use later in life (Cavalli and Cservenka 2020; Squeglia et al. 2014). Alternatively, it is well established in the literature that cannabis use acutely affects cognition, namely verbal learning, memory, executive functioning, cognitive flexibility, attention, and working memory (Broyd et al. 2016; Dellazizzo et al. 2022; Duperrouzel et al. 2020; Gonzalez et al. 2017). However, its long-term effects remain unclear (Dellazizzo et al. 2022). Meta analyses provide evidence for both cognitive recovery within a month of abstinence (Duperrouzel et al. 2020) and persisting effects (Broyd et al. 2016). Yet, cognitive recovery after sustained abstinence is found in adolescents (Lorenzetti et al. 2016) with effects being limited to a few days after use (Ellingson et al. 2021; Lorenzetti et al. 2016).

Finally, psychological, neurocognitive and brain changes during adolescence may play a causal or modulatory role in psychopathology (Schumann et al. 2010). Indeed, the endocannabinoid system is implicated in stress and anxiety regulation, particularly through its actions on corticolimbic structures. The changes these regions undergo during development put adolescents at increased risk for emotional and anxiety disorders (Meyer et al. 2018). The literature suggests that externalizing problems (i.e., when maladjustment is expressed mostly outward, e.g., conduct problems) likely precede cannabis use (e.g., Blair 2020; Farmer et al. 2015; Griffith-Lendering et al. 2011; Oshri et al. 2011), whereas associations with internalizing symptoms (i.e., when maladjustment is expressed mostly inward, e.g., emotional symptoms) have been weaker and more inconsistent (Griffith-Lendering et al. 2011).

Overall, further investigations employing longitudinal designs are necessary to address inconsistent findings. Variations in cannabis use patterns likely influence these discrepant results. Indeed, it is expected that light/occasional (i.e., weekly, or monthly) and heavy (i.e., daily, or near-daily) use during adolescence could lead to disparate outcomes later in life. Most adolescents engage in infrequent cannabis use, with non-disordered cannabis use being four times more prevalent than instances of Cannabis Use Disorder (Degenhardt et al. 2010; Sultan et al. 2023). As such, it is important to examine the neurocognitive and psychological outcomes among adolescents who escalate to heavier cannabis consumption as well as those who do not. For this purpose, we used archival data from a large longitudinal cohort, the IMAGEN study (Schumann et al. 2010). IMAGEN’s participants who used cannabis were mostly characterized by a low-to-moderate frequency of cannabis use (the percentage of participants reporting heavy use ranged from 22.8%—28.1%). This data was analyzed in an attempt to provide evidence regarding the following questions: (Q1) Do preexisting differences in reward-related brain activity, psychopathology, and cognitive functioning predict cannabis use initiation?; (Q2) Can cannabis use lead to impairments in these levels of functioning?; (Q2.1) If it does, do the disrupted levels of functioning recover with abstinence?

Given the current state of the literature, it is clearly challenging to establish robust hypotheses. Consequently, we adopted mainly an exploratory approach; although we anticipated that: (H1) externalizing psychopathology precedes cannabis use and (H2) cannabis-related cognitive impairments, if present, are expected to subside after a ≥ 1-month period of abstinence.

## Methods and materials

### Participants

#### IMAGEN sampling procedures

At baseline, 2341 adolescents (*Mage* = 14.33, *SDage* = 0.89; 48.4% female) were recruited at 8 sites in England, Ireland, France, and Germany. At each site, local ethics committees approved the protocol, and participants and legal guardians provided written informed consent. Participants completed a comprehensive test battery when they were, on average, 14 (baseline; BL), 19 (Follow-Up 1; FU1), and 22 years-old (Follow-Up 2; FU2). They were asked to abstain from caffeine, alcohol, and other drugs 24 h prior to testing. For further details on recruitment, assessment procedures, and IMAGEN’s exclusion criteria see Schumann et al. (2010).

#### Current sample

For inclusion in the current work participants had to report no (or low risk) alcohol use and nicotine dependence at BL. Exclusion criteria at BL also included: (a) having used a specific illicit substance > 2 times in their lifetime; (b) reporting > 8 total uses of illicit substances in their lifetime; and (c) reporting the use of “relevin” (i.e., fictitious substance). A total of 1946 drug-naïve participants (*Mage* = 14.39 years, *SDage* = 0.40; 51.2% female) were eligible for inclusion at BL. The control group (i.e., CON) was defined as the participants that maintained these criteria throughout all timepoints, resulting in 326 non-users (67.8% female).

At FU1, individuals who used cannabis reported using cannabis ≥ 6 times in the previous year and ≥ 1 time in the previous month. Cannabis was required to be the main substance they used during their lifetime. Participants with a possible alcohol dependency were excluded. At FU1, we identified 164 individuals who used cannabis (i.e., CAN group; 31.1% female).

From these, 57 (22.8% female) still used cannabis at FU2, while 19 (36.8% female) were cannabis abstinent for ≥ 1 months (i.e., ABS group). Inclusion criteria for the ABS group were: (a) being classified as CAN at FU1; (b) having used cannabis ≥ 20 times in their lifetime (cannabis being the main substance they used); and (c) not having used cannabis in the previous month.

### Questionnaires

**Fig. 1:**
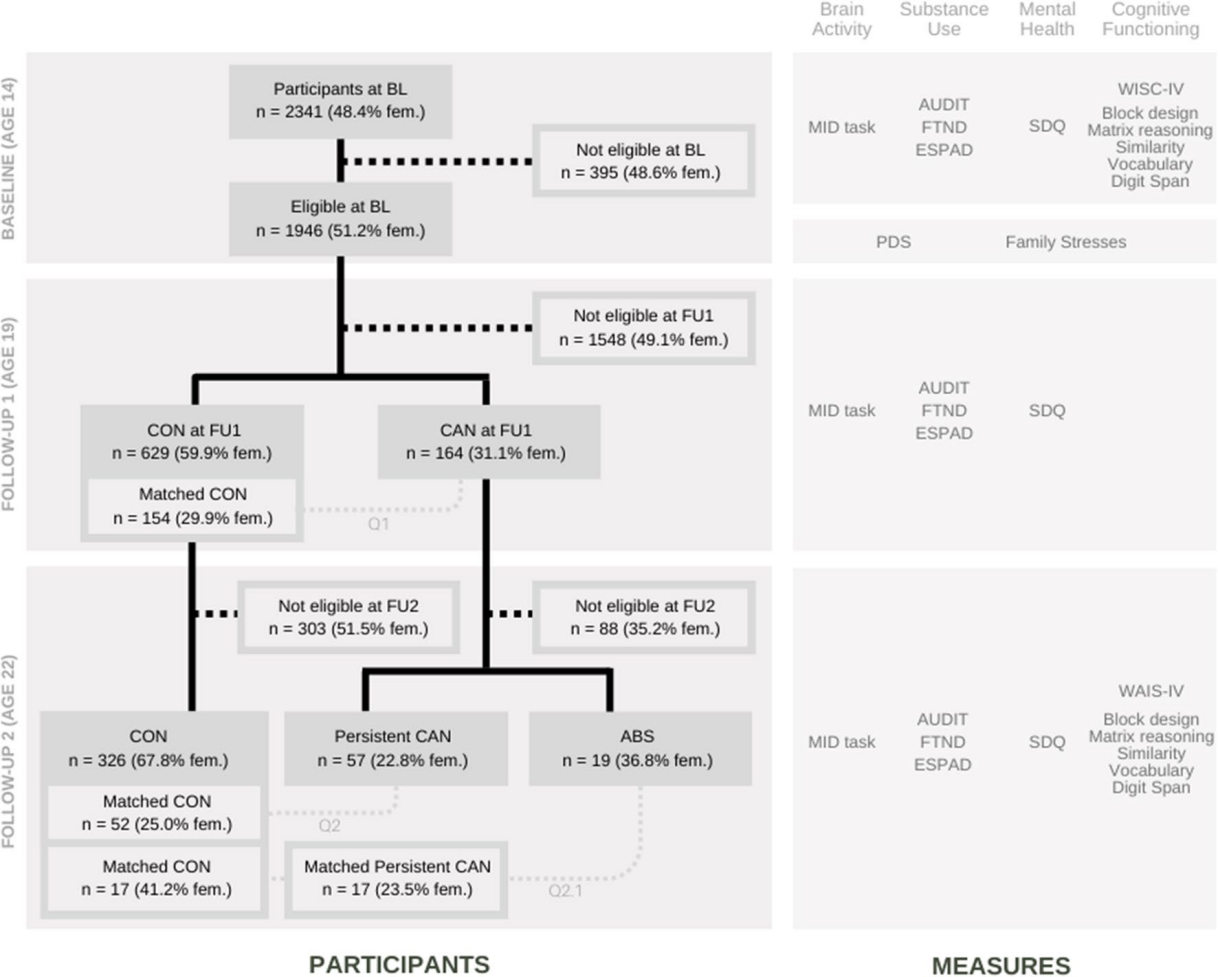
On the left: Flowchart of participant selection. On the right: measures administered at each timepoint to assess: (1) Brain activity: the Monetary Incentive Delay (MID) task; (2) Substance use: the European School Survey Project on Alcohol and Other Drugs (ESPAD), the Alcohol Use Disorders Identification Test (AUDIT), and the Fagerström Test for Nicotine Dependence (FTND); (3) Mental health: the Strengths and Difficulties Questionnaire (SDQ; which includes the emotional symptoms, peer problems, conduct problems, hyperactivity/inattention, and prosocial behavior subscales); and (4) Cognitive functioning: Wechsler Intelligence Scale for Children (WISC-IV) and for Adults (WAIS-IV)

At all timepoints (cf. Fig. 1 and Supplementary Materials), participants completed the following measures (a) substance use: the European School Survey Project on Alcohol and Other Drugs (ESPAD; Hibell et al. 2012), the Alcohol Use Disorders Identification Test (AUDIT; Saunders et al. 1993), the Fagerström Test for Nicotine Dependence (FTND; Heatherton et al. 1991); (b) psychopathology: the Strengths and Difficulties Questionnaire (SDQ; Goodman 1997); and (c) cognition: the block design, matrix reasoning, similarity, vocabulary, and digit span subtests of the Wechsler Intelligence Scale for Children (WISC-IV at BL) and for Adults (WAIS-IV at FU2) (Grizzle 2011; Wechsler 2008). Finally, to match participants on pubertal development and socioeconomic status (BL), the Puberty Development Scale (PDS; Petersen et al. 1988) and a scale assessing family stresses (Goodman et al. 2000) were administered.

### Experimental task

**Fig. 2:**
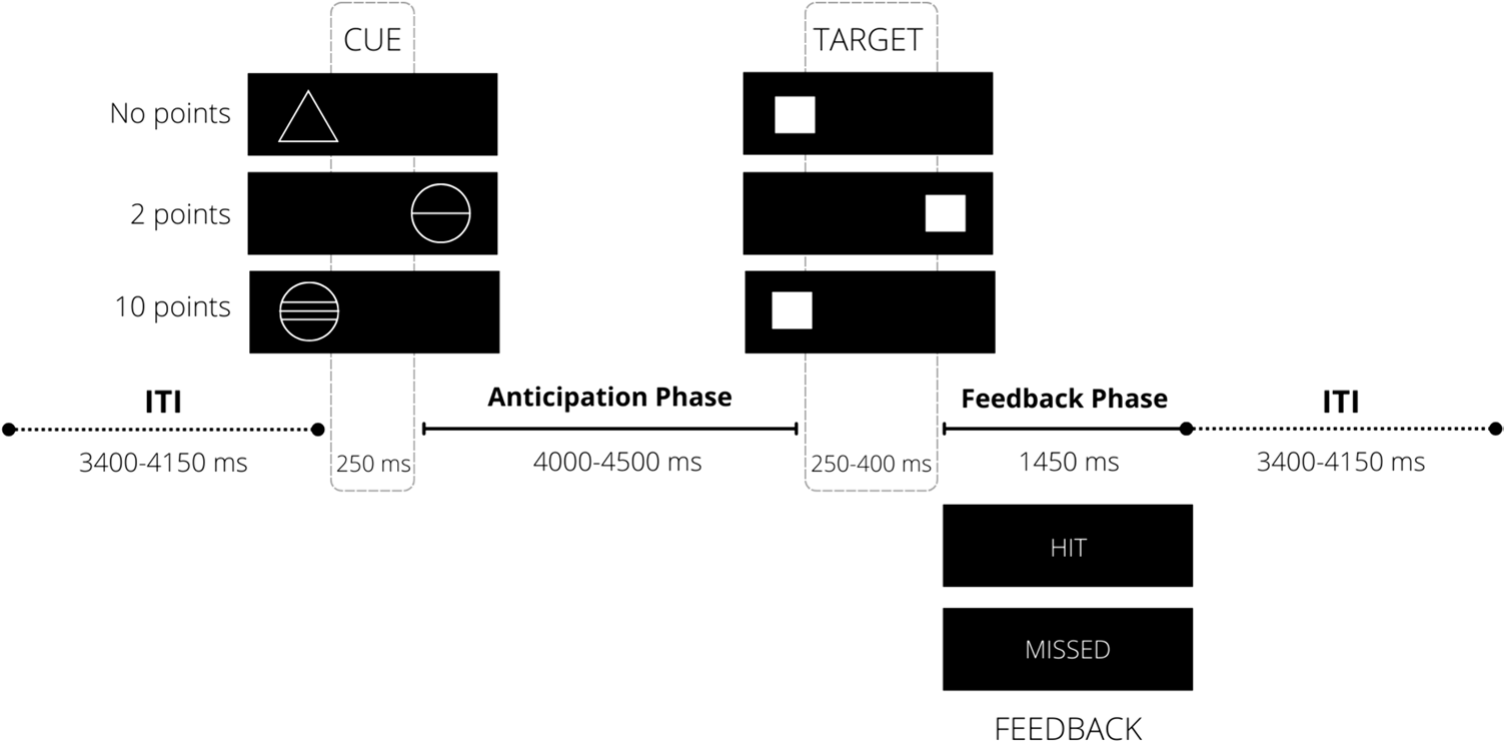
Graphical representation of the MID task (adapted from IMAGEN reports). *Note:* ITI = Intertrial Interval

At all timepoints, participants completed a modified version of the MID task (11 min at BL and 7 min at FU) with a 3-min practice block outside the fMRI scanner (Fig. 2). At the beginning of each trial, participants see a cue, which is followed by a target. They must respond as quickly as possible to targets by pressing a button. A successful trial (i.e., hit) occurs when the participant responds while the target is on the screen. Three types of cues inform the participants about the possibility of winning 2, 10, or no points (each condition was presented a third of the total trials). About 1.5 s after reacting, participants receive feedback on their performance (hit or missed). A jittered intertrial interval was used (3400–4150 ms). A tracking algorithm ensured that all participants had a success rate of ~ 66% by adapting the duration of the target throughout the task (between 100–300 ms). To enhance motivation, participants were informed that they would receive a sweet for every 5 points.

### fMRI data acquisition and processing

fMRI data acquisition and processing follow IMAGEN’s procedures (Schumann et al. 2010; Supplementary Materials). For quality control purposes we retrieved the head motion parameters of each participant. Participants with a mean framewise displacement > 0.5 mm were excluded (Power et al. 2012).

**Fig. 3:**
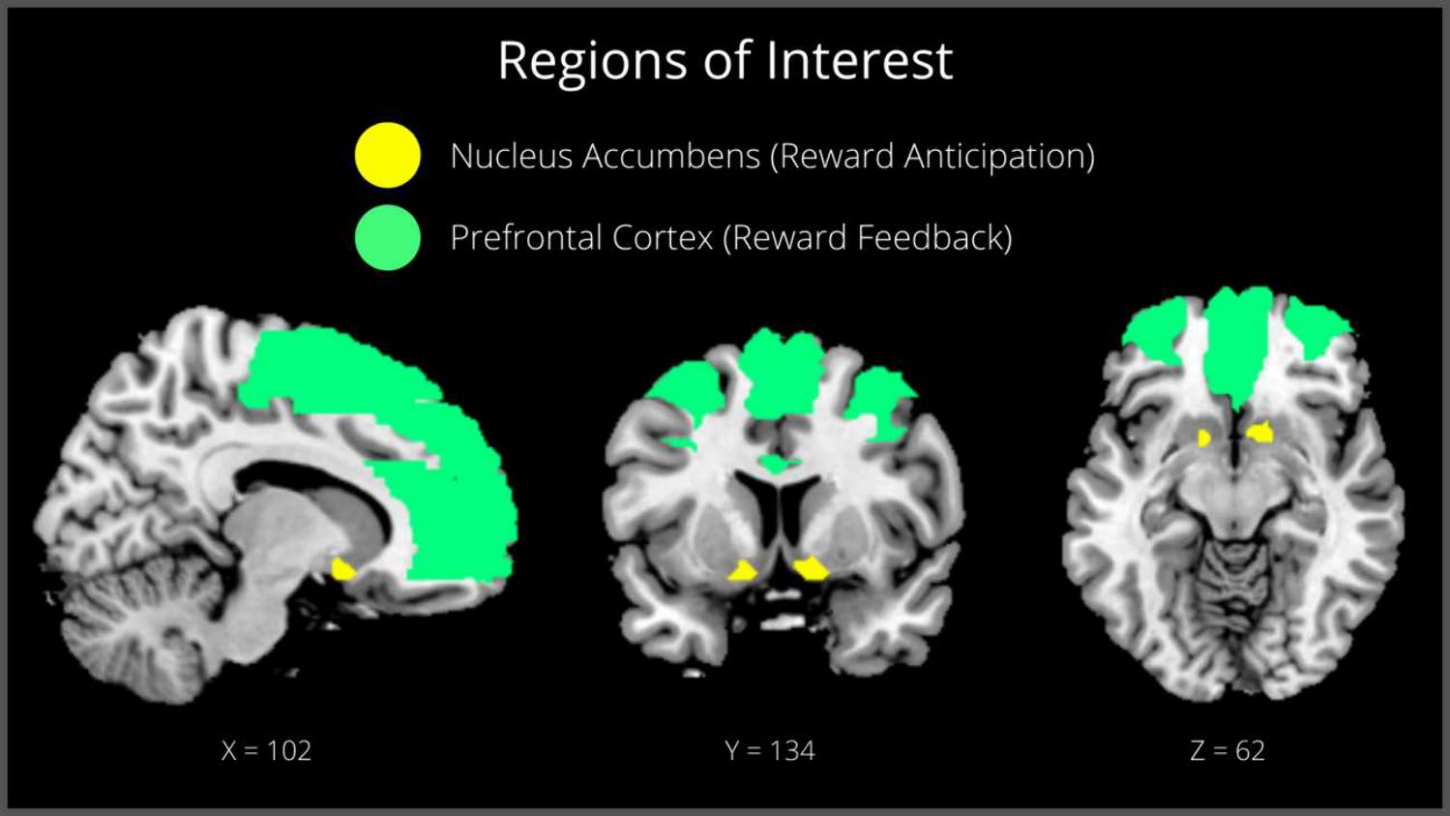
ROI analyses for reward anticipation—NAc (right and left NAc; 127 voxels)—and feedback—PFC (frontal mid left and right, frontal mid orb left and right, supp motor area left and right, frontal sup medial left and right, cingulum anterior left and right; 25 802 voxels). Shown in Montreal Neurological Institute (MNI) standard space and defined using the WFU PickAtlas tool

We focused on brain responses during gain anticipation (of both large and small gains) and reward feedback for successful (hits) and unsuccessful (missed) trials, separately. This resulted in four contrasts: gain anticipation > neutral (hit and missed trials separately); hit feedback on gain trial > feedback on neutral trial; missed feedback on gain trial > feedback on neutral trial. We used the WFU PickAtlas tool (52; https://www.nitrc.org/projects/wfu_pickatlas/) to create the masks for the Regions of Interest (ROI) analyses. For the anticipation phase we created a mask with the right and left NAc, and for the feedback phase we included prefrontal areas (i.e., bilateral: middle frontal gyrus, middle frontal gyrus in the orbitofrontal cortex, medial part of the superior frontal gyrus, supplementary motor area, anterior cingulate gyrus) (Fig. 3).

### Statistical analyses

Statistical analyses were performed using SPSS 28 (IBM Corp., Armonk, NY, USA) and SPM12 (MATLAB R2022b toolbox).

Propensity Score Matching was performed to balance group sizes and match participants on baseline age, sex, pubertal development, language, and socioeconomic status.

To address if baseline (14 years) characteristics predict cannabis use at 19 years (Q1), the 164 participants that engaged in cannabis use at 19 years (CAN) and matched non-users (CON) were included in four logistic regression analyses. Firstly, we extracted standardized beta-weights, representative of the mean BOLD response within the ROIs, and included them in two regression models as predictors: NAc activation during reward anticipation (2 predictors: missed and hit trials) and PFC activation during feedback processing (2 predictors: missed and hit trials). Then, each WISC-IV subtest score and the SDQ subscales’ scores (introduced in three blocks: 1st, conduct problems and hyperactivity/inattention symptoms, i.e., externalizing psychopathology; 2nd, emotional symptoms, i.e., internalizing psychopathology; and 3rd, peer problems and prosocial behavior) were included in two other logistic regressions.

To address differences between CAN and CON (Q2), a subsample of 57 persistent CAN and their matched CON were compared on SDQ and WISC/WAIS scores. We conducted repeated measures ANOVAs with *Time* (BL, FU1, FU2) and *Group* (persistent CAN, CON) and their interaction as factors, in separate models for each SDQ subscale and each WISC/WAIS subtest. Considering our goal of assessing group differences over time, we only focused on the pairwise comparisons of the Time*Group interaction factor. Similarly, using SPM’s full factorial design, we defined four models with two factors (*Group* and *Time*) to compare brain activity in the contrasts of interest.

Finally, on the measures in which significant group differences were found, repeated measures ANOVAs were conducted on a subsample of 19 individuals who used cannabis at 19 years but were abstinent at 22 years (ABS), their matched persistent CAN, and CON to assess potential recovery with abstinence (Q2.1).

To correct for multiple comparisons in self-report and neuropsychological indicators, the statistical significance threshold was determined using the two-stage False Discovery Rate (FDR) proposed by Benjamini et al. (2006) (max. = 0.05). The threshold for statistical significance was set at α = 0.014 (FDR-corrected *p*-values available in Supplementary Materials) (Pike 2011). Regarding neuroimaging data, a FWE-corrected threshold (*pFEW* < 0.05) and a cluster-extended threshold of 20 voxels were defined for identifying statistically significant differences in BOLD responses. The models were run for whole-brain and ROI analyses.

Effect sizes were calculated using Cohens’ *d*, eta squared (*η*^*2*^), and Cramer’s V for the t-tests, ANOVAs, and chi-square tests, respectively.

## Results

Full reports of the statistical analyses and missing data are available in the Supplementary Materials.

### Q1: Baseline predictors of cannabis use initiation at age 19

#### Propensity Score Matching and group characteristics

The 164 participants (*Mage* = 14.34, *SDage* = 0.40) who started to use cannabis at age 19 (i.e., CAN group) were successfully matched to 154 CON (*Mage* = 14.31, *SDage* = 0.40) at baseline. Table 1 reports the descriptive statistics and group comparisons.^1^

**Table 1:** Baseline (14 years) sociodemographic, neuropsychological, and fMRI data descriptive statistics of adolescents that would engage in cannabis use at 19 years (CAN at FU1) and matched controls (CON) and their cannabis use frequency at FU1 (19 years)

	CAN at FU1 (*n* = 164)	CON (*n* = 154)	Group Comparisons		
			*t*_(df)_ / *X*^*2*^	*P*	*d/ V*
**Age** *M* (*SD*)	14.34 (0.40)	14.31 (0.40)	-0.737_(316)_	.462	0.08
[Min., Max.]	[13.00, 15.38]	[13.33, 15.54]			
**Sex** *n (%)*			0.056_(1)_	.812	0.01
Male	113 (68.9%)	108 (70.1%)			
Female	51 (31.1%)	46 (29.9%)			
**Pubertal Development** *M (SD)*					
Male	2.63 (0.58)	2.54 (0.53)	-1.184_(222)_	.238	0.16
Female	4.10 (0.22)	4.10 (0.24)	0.080_(84)_	.937	0.02
**Language** (%)			2.953_(2)_	.228	0.10
French	39 (%)	34 (%)			
English	58 (%)	43 (%)			
German	67 (%)	77 (%)			
**Socioeconomic status** *M (SD)*	2.88 (2.39)	2.84 (3.20)	-0.119_(283)_	.905	0.01
**SDQ (self-report)** *M (SD)*					
Total difficulties	10.06 (4.73)	9.66 (5.25)	-0.724_(316)_	.470	0.08
Emotional Symptoms	2.36 (2.09)	2.17 (2.01)	-0.829_(316)_	.408	0.09
Conduct Problems	2.38 (1.55)	1.82 (1.46)	-3.270_(316)_	.001	0.37
Hyperactivity/ Inattention	3.74 (2.07)	3.56 (2.23)	-0.742_(316)_	.459	0.08
Peer Problems	1.58 (1.54)	2.10 (1.89)	2.687_(316)_	.008	0.30
Prosocial	7.39 (1.65)	7.71 (1.83)	1.630_(316)_	.104	0.18
WISC-IV* *M (SD)*					
Block Design	0.05 (0.95)	0.21 (0.88)	1.513_(299)_	.131	0.18
Digit Span	0.11 (0.95)	0.07 (0.91)	-0.375_(305)_	.708	0.04
Matrix Reasoning	0.06 (0.97)	0.02 (0.94)	-0.414_(299)_	.679	0.05
Similarities	0.26 (0.83)	0.10 (0.84)	-1.679_(300)_	.094	0.19
Vocabulary	0.18 (0.89)	0.01 (0.86)	-1.650_(399)_	.100	0.19
**fMRI** *M (SD)*					
Mean FD	0.22 (0.9)	0.22 (0.09)	-0.126_(255)_	.900	0.09
NAc Anticip Hit**	0.93 (1.07)	0.99 (0.91)	0.396_(233)_	.693	0.99
NAc Anticip Missed**	0.75 (0.89)	0.69 (1.04)	-0.457_(243)_	.648	0.96
PFC Feedback Hit**	0.05 (0.33)	0.08 (0.36)	0.598_(227)_	.550	0.34
PFC Feedback Missed**	-0.02 (0.36)	-0.09 (0.37)	-1.494_(228)_	.137	0.36
**Cannabis use at 19 years-old**					
**Lifetime** *n (%)*					
0	0	115 (74.68%)			
1–2	0	39 (25.32%)			
3–5	1 (0.61%)	0			
6–9	7 (4.27%)	0			
10–19	23 (14.02%)	0			
20–39	33 (20.12%)	0			
≥ 40	100 (60.98%)	0			
**Previous year** *n (%)*					
0	0	136 (88.31%)			
1–2	0	18 (11.69%)			
3–5	0	0			
6–9	27 (16.46%)	0			
10–19	38 (23.17%)	0			
20–39	28 (17.07%)	0			
≥ 40	71 (43.29%)	0			
**Previous month** *n (%)*					
0	0	154 (100%)			
1–2	49 (29.88%)	0			
3–5	30 (18.29%)	0			
6–9	20 (12.20%)	0			
10–19	32 (19.51%)	0			
20–39	20 (12.20%)	0			
≥ 40	13 (7.93%)	0			
**Previous week** *n (%)*					
0	47 (28.66%)	154 (100%)			
1–2	55 (33.54%)	0			
3–5	26 (15.85%)	0			
6–9	18 (10.98%)	0			
10–19	11 (6.71%)	0			
20–39	5 (3.05%)	0			
≥ 40	2 (1.22%)	0			

*SDQ* = *strengths and difficulties questionnaire; WISC* = *Wechsler Intelligence Scale for Children – Fourth Edition (WISC-IV); fMRI* = *functional Magnetic Resonance Imaging; FD* = *Framewise Displacement; NAc* = *Nucleus accumbens; Anticip* = *Anticipation; PFC* = *Prefrontal Cortex*

^*^Z-scores

^**^Standardized beta-weights

#### Binary logistic regressions

##### NAc activation during the anticipation phase.^1^

The model was non-significant, *χ*^*2*^(2) = 0.70, *p* = 0.705.

##### PFC activation during the feedback phase.^1^

The model was non-significant, *χ*^*2*^(2) = 2.66, *p* = 0.264.

##### Cognitive functions

The model was non-significant, *χ*^*2*^(5) = 8.79, *p* = 0.118.

##### Psychopathology

A statistically significant model, *χ*^*2*^(5) = 26.71, *p* < 0.001, explained 10.7% (Nagelkerke *R*^2^) of the variance of CAN membership, and correctly classified 61.9% of the cases. Specifically, higher conduct problems scores (OR = 1.35, *p* = 0.001) and lower peer problems scores (OR = 0.75, *p* < 0.001) at 14 years were associated with a greater likelihood of using cannabis at 19 years.

### Q2: Comparing participants who use cannabis and non-users

#### Propensity score matching analyses and group characteristics

The 57 persistent CAN at age 22 (*Mage* = 14.29, *SDage* = 0.39) were successfully matched to 52 CON (*M age* = 14.34, *SDage* = 0.42) on their baseline characteristics.

#### Repeated-Measures ANOVAs

##### fMRI

For both whole-brain and ROI analyses (NAc and PFC) no clusters yielded significant effects of *group* or group*time interaction (for neither missed nor hit conditions, all *pFWE* > 0.05).^2^

##### Cognitive functions

There were no statistically significant effects of *group*, or group*time interaction (all *p* > 0.06).

##### Psychopathology

We found statistically significant between-subjects effects for conduct problems, *F*(1, 106) = 13.27, *p* < 0.001, *η*^*2*^ = 0.11, and hyperactivity/inattention, *F*(1, 106) = 9.04, *p* = 0.003, *η*^*2*^ = 0.08. The persistent CAN group had higher scores on the conduct problems scale at 14 (*p* < 0.001) and 19 (*p* = 0.012) years-old; also, higher hyperactivity/inattention scores at 19 years-old (*p* = 0.008) (Fig. 3).

### Q.2.1: Recovery with at least one month of abstinence

#### Propensity score matching and group characteristics

Nineteen ABS at 22 years (*Mage* = 14.34, *SDage* = 0.48; 36.8% female) were successfully matched to 17 CON (*Mage* = 14.31, *SDage* = 0.40), and 17 persistent CAN (*Mage* = 14.37, *SDage* = 0.44) on their baseline characteristics. Nine ABS (47.4%) had not used cannabis for over a year and none in the previous month.

#### Repeated-measures ANOVAs

##### Psychopathology

**Fig. 4:**
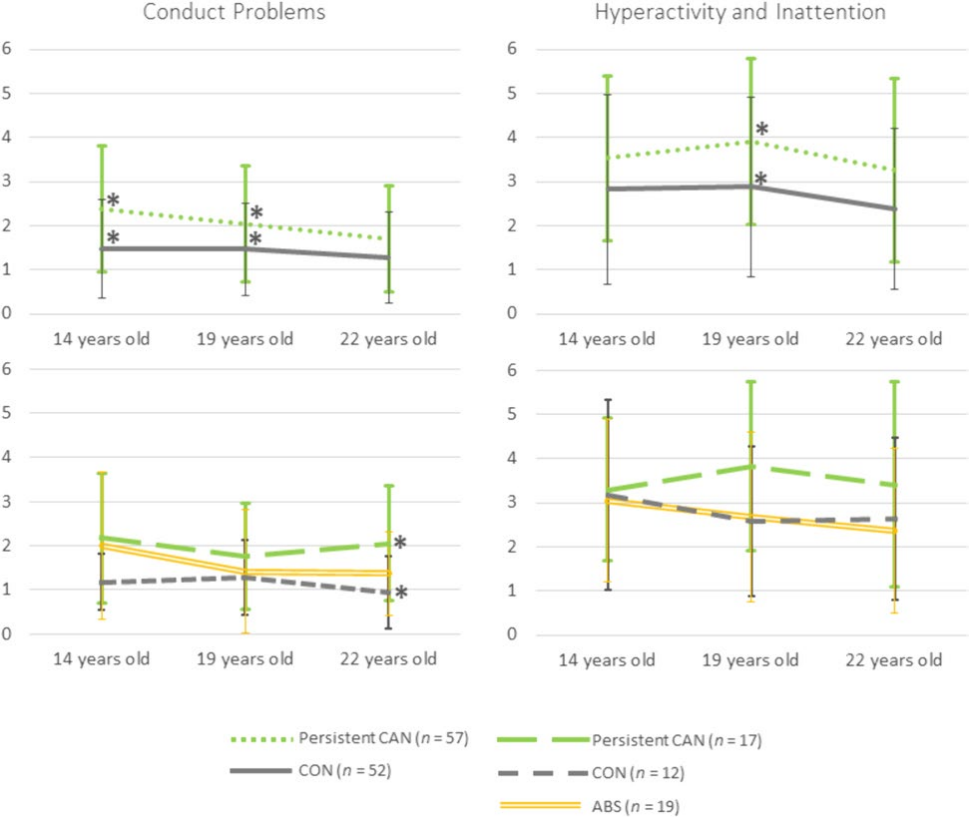
Conduct problems and hyperactivity/inattention symptoms’ scores (scores range: 0–10) of participants that persistently used cannabis (CAN), participants that used cannabis at 19 years old but were abstinent at 22 years old (ABS), and participants that did not use cannabis (CON)

The persistent CAN subgroup reported higher conduct problems scores (*M* = 1.06, *SD* = 0.25) than CON (*M* = 0.94, *SD* = 0.25) at 22 years (*p* = 0.009), *F*(1, 50) = 4.11, *p* = 0.022, *η*^*2*^ = 0.14. ABS did not differ from persistent CAN (all *p* > 0.05) nor CON (all *p* > 0.07). No group differences were found for hyperactivity/inattention symptoms (*p* = 0.246) (Fig. 4).

## Discussion

The goal of the current work was to explore differences between people who used cannabis and non-users in reward-related brain activity, psychopathology, and cognitive functioning before and after cannabis use onset. For this purpose, we used archival data of adolescent participants from the IMAGEN longitudinal cohort-study (Schumann et al. 2010). Most CAN participants reported light cannabis use. Studying the effects of such levels of use in the adolescent brain is of paramount importance given that most adolescents who use cannabis do it infrequently and few investigations have examined the long-term outcomes associated with occasional use (Degenhardt et al. 2010; Sultan et al. 2023). For instance, according to the 2019 European School Survey Project on Alcohol and Other Drugs, 88.32% of teenagers who reported using cannabis in the previous month described non-daily use (for more information see: http://espad.org/).

Regarding preexisting differences, results partially support H1, which posited that externalizing psychopathology would precede cannabis use and be more prevalent in the CAN group. Scoring higher on conduct problems and lower on peer problems was associated with a greater likelihood of belonging to the CAN group. Neither internalizing psychopathological dimensions (i.e., emotional symptoms), cognitive functioning, NAc, nor PFC activity in the MID task predicted future cannabis use.

Similarly, after cannabis use initiation, light CAN and CON did not differ in internalizing psychopathology, cognitive functioning, or brain activity (Q2). However, the CAN group scored higher on conduct problems and hyperactivity and inattention symptoms at 19 years. Moreover, at 22 years, persistent CAN, but not ABS, exhibited significantly higher conduct problems than CON (Q2.1). Due to the absence of significant differences in cognitive functioning and brain activity, we did not test the hypothesis concerning recovery with abstinence (H2). Next, we will discuss the main findings.

### Reward processing

In a sample of 318 cannabis-naïve 14-year-olds, neither NAc (reward anticipation) nor PFC activity (feedback processing) predicted cannabis use at 19 years-old. This suggests the absence of preexisting differences in the reward-related brain activity of adolescent who will use cannabis in the future.^3^ Importantly, the current sample was mostly characterized by a low-to-moderate pattern of cannabis use, and previous works reporting similar frequencies of use (e.g., Skumlien et al. 2022) have concluded that such levels of cannabis use are probably not associated with disruptions in reward-related brain activity.

We also found no differences in BOLD responses in the assessed brain structures in individuals who still occasionally used cannabis at 22 years. In line with our results, previous cross-sectional studies found no group differences in whole-brain analysis, and/or NAc activity during reward anticipation, and/or PFC activity during reward feedback (e.g., Filbey et al. 2013; Jager et al. 2013; Karoly et al. 2015; Nestor et al. 2020; Skumlien et al. 2022; Yip et al. 2014). It should be noted, however, that the MID task version we used did not include a loss condition. Some of the studies that did not find group differences in brain activity during gain-related feedback found them for loss-related feedback (Filbey et al. 2013; Yip et al. 2014).

Regarding longitudinal designs, one study (Martz et al. 2016) in a young sample indicated that long-term disruptions in neural circuits associated with reward anticipation, namely blunted NAc activity at a 2 year and a 4 year follow up, may be induced by cannabis use rather than a risk factor for cannabis use initiation. In this study, the findings—that differ from ours—may indicate that the sample was more susceptible to cannabis-induced sequels in reward-related regions because the participants were considered high-risk and already had a history of cannabis use at baseline. Indeed, genetics and epigenetics can play a critical (and complex) role in cannabis use (Dennen et al. 2022). Some potential explanations are: (a) genetic predisposition for higher reward processing in high-risk samples; (b) neural changes prompted by adverse life experiences endured by children with parents with history of substance abuse; (c) an interaction between both heritable and environmental factors; or (d) cannabis use may be a coping mechanism to deal with negative emotionality, which will then be exacerbated by the effects of cannabis on the dopaminergic reward system (Martz et al. 2016). The current study provides some insights on this latter point by showing that internalizing symptoms, specifically emotional symptoms, did not explain the onset of cannabis use.

### Cognitive functioning

We did not find evidence of cognitive impairment in light CAN neither before nor after cannabis use onset. Indeed, it is suggested that despite the broad association that may exist between adolescent cannabis use and neurocognitive impairment, these effects appear to be minor and may not be clinically significant (e.g., Ellingson et al. 2021; Lorenzetti et al. 2016).

Nonetheless, some variables may moderate these findings. A greater representation of light CAN may be one of these variables, since a previous IMAGEN study (Wendel et al. 2021) also found no longitudinal effects of cannabis on attention, working memory, and short-term memory from 14 to 19 years old. There were also no baseline differences between future CAN and CON. Conversely, another longitudinal study (7 to 45 years-old) observed that long-term cannabis use was associated with cognitive deficits only in midlife and in a dose-dependent manner; such that people who used cannabis heavily showed greater decline later in life (Meier et al. 2022). This raises the possibility that cognitive impairments in the heavier users may come through later stages of development and not during the age ranges we assessed.

Overall, future longitudinal studies should oversample participants with higher frequency of cannabis use and follow them through midlife to re-evaluate this pattern of findings.

### Psychopathology

Externalizing problems predicted future cannabis use. Specifically, conduct problems in 14-year-old drug-naïve adolescents predicted a greater likelihood of transitioning to cannabis use within five years. This is consistent with previous works (e.g., Blair 2020; Farmer et al. 2015; Griffith-Lendering et al. 2011; Oshri et al. 2011) and supports the idea that conduct problems may be a gateway to substance use. Interestingly, having peer problems negatively predicted the CAN status at 19 years. This seems to suggest that, under certain circumstances, being more sociable and better integrated with peers may increase the likelihood of becoming a CAN. This may be particularly true when it comes to exposure to peers with conduct problems (Van Ryzin et al. 2012). Social context is a critical aspect for substance use, particularly during adolescence, when peers exert a socializing influence and peer pressure, namely on individual substance use (Andrews et al. 2002). Alternatively, adolescents may bond more strongly with peers with whom they best identify with and that already share similar interest in substance use (Andrews et al. 2002). Curiously, previous research has also found that supportive peer relationships were associated with a higher risk for cannabis use and psychopathological symptoms among adolescents with lower family support (Moore et al. 2018). These dimensions may help to identify high-risk users in cannabis use prevention programs.

Notwithstanding the overall decline in conduct problems with age, the persistent CAN group exhibited more enduring conduct problems than CON at age 22. Regarding participants who used cannabis at 19 years but were abstinent at 22 years-old, they did not differ from persistent CAN nor CON. Indeed, the literature suggests that, for most individuals, displays of problematic behavior during adolescence do not become chronic (Monahan et al. 2014). Cannabis use may be a teenagers’ way of proclaiming their self-determination, feeling more mature, or even fit in their peer group (Monahan et al. 2014). As they enter young adulthood and these social forces lose their influence, they abstain from cannabis use. As reported in the Supplementary Material, the ABS group already reported a smaller frequency of cannabis use at 19 years compared to the future persistent CAN, further supporting the possibility that lower levels of conduct problems may be associated with short-term, less frequent cannabis use, whereas chronic cannabis use may arise in teenagers with increased conduct problems. However, it is relevant to note that, in the current sample, conduct problems’ scores were relatively small, even in the persistent CAN group. Future research examining the distinct developmental pathways of cannabis users with a wider range of conduct problems’ severity are needed to support this assumption and inform preventive measures.

Hyperactivity and inattention symptoms at 14 years did not predict future cannabis use, but the CAN group at 19 years had higher symptoms than CON. Previous cross-sectional studies have already described this association (e.g., Petker et al. 2020). Our results suggest that, in community samples of adolescents (for clinical samples see Francisco et al. 2023), cannabis use may lead to hyperactivity and inattention symptoms, and not the other way around. Unlike conduct problems, these symptoms do not appear to be a strong risk factor for cannabis use, probably lacking clinical significance and subsiding with time in adolescents who engage in low-frequency cannabis use. However, it should be noted that the presence of such symptoms during adolescence may affect educational outcomes. Longitudinal studies have demonstrated that adolescents who use cannabis are less likely to complete high school than non-users, which can influence future income, occupation, and life chances (Lorenzetti et al. 2020).

Finally, the lack of differences regarding internalizing symptoms (specifically emotional symptoms) is not surprising as previous works have reported both weak associations with cannabis use and no evidence of them representing risk factors for cannabis use during adolescence or early adulthood (Farmer et al. 2015; Griffith-Lendering et al. 2011). Overall, the current results support the notion that cannabis use is more reliably associated with externalizing psychopathology than to internalizing psychopathology.

### Limitations and future directions

Some limitations of the current work must be noted. First, although our study's sample size exceeds that of many other studies, a larger sample would have been ideal for more robust statistical analysis and generalization of findings. It is possible that our group comparisons are underpowered, thus replication studies will be essential to confirm our findings. This is particularly true for the assessment of cognitive functions, given that there were only two moments of assessment (14 and 22 years) and many participants did not complete the subtests at both timepoints. Additionally, some relevant cognitive domains were not specifically assessed. Consequently, these results should be interpreted with particular caution and not generalized to other cognitive domains. Second, due to the exploratory nature of the current study we decided to report pairwise comparisons even in the absence of a significant main effect of Time*Group interaction. As such, more longitudinal studies are crucial to corroborate the current results. Third, the CAN group in this study reported a relatively low frequency of use; only 22.8% and 28.1% of CAN reported daily or near daily use at 19 and 22 years, respectively. Having a greater representation of heavy CAN would increase the generalization of findings, even though the cannabis use patterns of our sample are comparable to those of previous works that have also found no group differences in reward-related brain activity (e.g., Karoly et al. 2015; Skumlien et al. 2022). Thus, the current findings may only reflect the effects experienced by low frequency CAN and not heavy users or individuals with Cannabis Use Disorder. Finally, when considering the association between conduct problems and cannabis in this sample, one cannot disregard the influence of the illegal status of the substance in the participants’ countries. Even if conduct problems precede cannabis use, the maintenance of these problems for as long as people use is inevitable given that only people who risk illegal behavior can access cannabis.

## Conclusion

The current study’s design allowed an examination of potential preexisting differences in brain activity, cognitive functioning, and psychological symptoms in a developmental sample of adolescents who would engage in light cannabis use in the future. We found no evidence of preexisting individual differences in reward processing or specific cognitive domains. However, cannabis-naïve adolescents with conduct problems and who were more socially engaged with their peers seem to be at a higher risk of taking part in persistent cannabis use in the future. Additionally, using cannabis during adolescence may result in the development of hyperactivity and inattention symptoms.

### Supplementary Information

Below is the link to the electronic supplementary material.

Supplementary file1 (DOCX 59 kb)

Supplementary file2 (XLSX 295 kb)

## Footnotes

1 We performed exploratory analyses to compare baseline brain activity of adolescents that would consume cannabis daily (or near-daily; i.e., ≥ 20 uses in the previous month) in the future with their matched controls. Independent samples t-test revealed no statistically significant group differences (see Table S4 in the Supplementary Material).

2 As requested by the reviewers, specific ROI analyses were further conducted on smaller structures, namely the ventromedial PFC and the orbitofrontal cortex. No clusters yielded significant effects of *group* or *group*time* interaction (*pFWE* > .05).

3 See, in the Supplementary Material, exploratory analyses with only the heavy CAN and their matched CON. No statistically significant differences were found.

## Declarations

Dr Banaschewski served in an advisory or consultancy role for eye level, Infectopharm, Lundbeck, Medice, Neurim Pharmaceuticals, Oberberg GmbH, Roche, and Takeda. He received conference support or speaker’s fee from Janssen, Medice and Takeda. He received royalties from Hogrefe, Kohlhammer, CIP Medien, Oxford University Press; the present work is unrelated to these relationships. Dr Barker has received honoraria from General Electric Healthcare for teaching on scanner programming courses. Dr Poustka served in an advisory or consultancy role for Roche and Viforpharm and received a speaker's fee from Shire. She received royalties from Hogrefe, Kohlhammer and Schattauer. The present work is unrelated to the above grants and relationships.
