# Supplementary material for: Light Cannabis Use and the Adolescent Brain: An 8-years Longitudinal Assessment of Mental Health, Cognition, and Reward Processing
Source: Psychopharmacology (Berl). 2024 Mar 26;241(7):1447–61. doi: 10.1007/s00213-024-06575-z (PMC11199211; doi:10.1007/s00213-024-06575-z)
Supplement: Supplementary file 1 — Supplementary file1 (DOCX 59 kb) [file 213_2024_6575_MOESM1_ESM.docx]

**Supplementary Materials**

**Extended Methods and Materials**

**Questionnaires**

All instruments were administered in the participants’ native language. Participants completed the ESPAD questionnaire (at all timepoints) (Hibell et al., 2012), which gathers detailed self-reported information regarding use (age of onset, weekly, monthly, yearly, and lifetime frequency of use) of the following substances: cannabis, alcohol, tobacco, amphetamines, methamphetamine, anabolic steroids, cocaine, crack, γ-hydroxybutyric acid (GHB), aerosols, ketamine, lysergic acid diethylamide (LSD), ecstasy, other hallucinogens, narcotics, sedatives, and heroin. Additionally, it includes control questions regarding a fictional substance (“*relevin*”). Frequency of use is measured in an ordinal scale: 0 uses, 1-2 uses, 3-5 uses, 6-9 uses, 10-19 uses, 20-39 uses, or ≥40 uses.

The Alcohol Use Disorders Identification Test (AUDIT; Saunders et al., 1993) and the Fagerström Test for Nicotine Dependence (FTND; Heatherton et al., 1991) were administered at all timepoints to assess alcohol and nicotine dependence. The AUDIT is a 10-item instrument that assesses alcohol dependence and total scores can range from 0-40, with higher scores reflecting greater likelihood of alcohol dependence (Cooke et al., 2019). The FTND is a 6-item self-report instrument that assesses intensity of nicotine addiction (Heatherton et al., 1991). Scores range from 0-10, with higher scores representing a more intense dependence on nicotine.

For assessing psychopathology we used the Strengths and Difficulties Questionnaire (SDQ; Goodman, 1997). This 25-item instrument assesses behavioral and emotional symptoms, namely: emotional symptoms, conduct problems, hyperactivity/inattention, peer relationship problems, and prosocial behavior (Goodman, 1997). The emotional symptoms subscale has been associated with internalizing psychopathology, whereas the conduct problems and hyperactivity/inattention scores have been associated with externalizing psychopathology (Goodman, 2001). Scores can range from 0-40 in the total difficulties scale and from 0-10 in the subscales. Higher scores represent worse outcomes, except for prosocial behavior. Both self-report (all timepoints) and parent (baseline) versions were administered.

The similarities, vocabulary, matrix reasoning, block design, and digit span subtests of the Wechsler Intelligence Scale for Children – Fourth Edition (WISC-IV) (Grizzle, 2011) and the Wechsler Intelligence Scale for Adults – Fourth Edition (WAIS-IV) (Wechsler, 2008) were administered at baseline and FU2, respectively. The similarities and vocabulary subtests represent a verbal comprehension index; while the similarities subtest considers higher-order executive functioning (e.g., verbal abstract reasoning, abstract thinking), the vocabulary subtest is more focused on language skills (e.g., knowledge of word meanings, verbal fluency). The matrix reasoning subtest measures non-verbal fluid reasoning. Similarly, the block design subtest measures abstract problem-solving abilities, visual abstract ability, and spatial analysis. Finally, the digit span subtest assesses working memory and attention (Figueiredo et al., 2020). Higher scores represent a better performance. Raw scores were transformed in z-scores to allow between-test comparisons.

The Puberty Development Scale (PDS; Petersen et al., 1988) and a family stresses scale (Goodman et al., 2000) were also administered at BL. The PDS is a 8-item self-report measure of adolescent physical development (higher scores indicate a more advanced developmental stage) (Petersen et al., 1988). The Family Stresses Scale (Goodman et al., 2000) is a 12-item questionnaire that was completed by the legal guardian of the participant. It evaluates economic status, parental relationships, working status, health, and addiction problems. Scores can range from 0 to 24, with higher scores representing increasingly concerning socioeconomic conditions. Baseline data from these measures will be used to match participants on pubertal development and socioeconomic status.

**fMRI data acquisition and preprocessing**

fMRI data was assessed as described by the IMAGEN consortium (Schumann et al., 2010). BOLD functional images were acquired with a gradient-echo planar imaging (EPI) sequence. Whole-body 3T magnetic resonance scanners were used at all sites using imaging sequences with the same parameters. Three hundred (at BL) or 191 (at FU1 and FU2) volumes with 40 slices (thickness: 2.4 mm; gap: 1 mm) were acquired using a gradient-echo T2*-weighted EPI sequence (TR = 2200 ms; TE = 30 ms; 64 x 64 in plane resolution; field of view = 220 x 220 mm). Anatomical reference included a gradient-echo sequence (TR = 6.8 ms; TE = 3.2 ms). A high-resolution T1 anatomical image (T1W magnetization prepared gradient echo sequence, MPRAGE) with 1×1×1 mm voxel size was also acquired for each participant.

SPM8 and SPM12 (Welcome Trust Centre for Neuroimaging, London, UK) were used for image preprocessing by the IMAGEN consortium. The pipeline is described elsewhere (Schumann et al., 2010).

SPM’s default hemodynamic response function (HRF) was used in the first-level general linear model, which included 12 regressors. Each of the following regressors was separately available for hit and miss trials: anticipation of large gain; anticipation of small gain; anticipation of no gain; large gain feedback; small gain feedback; and no gain feedback.

**Extended Results**

**Propensity Score Matching Analysis**

The propensity score matching analyses were performed on SPSS 28 using a match tolerance of 0.2 (0 = exact matches, 1 = any control could match any case). Participants were matched based on their baseline age, sex, pubertal development, language, and socioeconomic status. Table S1 shows the baseline sociodemographic characteristics of the controls and future cannabis users before the matching procedure.

**Table S1.**

*Sociodemographic characteristics of the participants before the matching procedures*

|  | | **Controls** (*n* = 629) | | | | **Cannabis users at 19 years** (*n* = 164) | | | |
| --- | --- | --- | --- | --- | --- | --- | --- | --- | --- |
|  |  | *M* | *SD* | *n* | % | *M* | *SD* | *n* | % |
| **Age** | | 14.40 | 0.41 |  |  | 14.34 | 0.40 |  |  |
| **Sex** | Male |  |  | 252 | 40.06% |  |  | 113 | 68.90% |
|  | Female |  |  | 377 | 59.94% |  |  | 51 | 31.10% |
| **PDS** Male | | 2.62 | 0.52 |  |  | 2.63 | 0.58 |  |  |
| Female | | 4.12 | 0.25 |  |  | 4.10 | 0.22 |  |  |
| **Socioeconomic status** | | 2.55 | 2.55 |  |  | 2.88 | 2.39 |  |  |
| **Language** | French |  |  | 76 | 12.08% |  |  | 39 | 23.78% |
|  | English |  |  | 202 | 32.11% |  |  | 58 | 35.37% |
|  | German |  |  | 351 | 55.80% |  |  | 67 | 40.85% |

**Baseline predictors of cannabis use at 19 years old (Q1)**

Only 151 cannabis users and 143 non-users had fMRI data at BL, and 15 users and 22 controls were excluded due to excessive head movement (FD > 0.5). Descriptive statistics of substance use patterns at baseline are presented in Table S2 and results from the Logistic Regressions are presented in Table S3.

| **Table S2.**  *Baseline (14 years) lifetime substance use of future cannabis users at 19 years old and matched controls* | | | | | |
| --- | --- | --- | --- | --- | --- |
|  | **Cannabis users at 19 years**  (*n* = 164) | **Controls**  (*n* = 154) | **Group Comparisons** | | |
|  |  |  | *t*_(df)_ / *X^2^* | *p* | *d/ V* |
| **Lifetime alcohol use** *(%)* |  |  | 29.47_(6)_ | <.001 | 0.30 |
| 0 | 32 (19.5%) | 55 (35.7%) |  |  |  |
| 1-2 | 38 (23.2%) | 45 (29.2%) |  |  |  |
| 3-5 | 24 (14.6%) | 27 (17.5%) |  |  |  |
| 6-9 | 27 (16.5%) | 6 (3.9%) |  |  |  |
| 10-19 | 25 (15.2%) | 14 (9.1%) |  |  |  |
| 20-39 | 20 (6.1%) | 6 (3.9%) |  |  |  |
| ≥40 | 8 (4.9%) | 1 (0.7%) |  |  |  |
| **AUDIT score** *M (SD)* | 1.32 (1.72) | 0.68 (1.15) | -3.88_(316)_ | <.001 | 0.44 |
| **Lifetime cigarette use** *(%)* |  |  | 27.70_(6)_ | <.001 | 0.26 |
| 0 | 105 (64.0%) | 134 (87.0%) |  |  |  |
| 1-2 | 22 (13.4%) | 14 (9.1%) |  |  |  |
| 3-5 | 9 (5.5%) | 1 (0.7%) |  |  |  |
| 6-9 | 8 (4.9%) | 1 (0.7%) |  |  |  |
| 10-19 | 7 (4.3%) | 2 (1.3%) |  |  |  |
| 20-39 | 6 (3.7%) | 1 (0.7%) |  |  |  |
| ≥40 | 7 (4.3%) | 1 (0.7%) |  |  |  |
| **FTND score** *M (SD)* | 0.04 (0.26) | 0.00 (0.00) | -2.07_(316)_ | <.001 | 0.23 |
| **Lifetime cannabis use** *(%)* |  |  |  |  |  |
| 0 | 150 (91.5%) | 153 (99.4%) |  |  |  |
| 1-2 | 14 (8.5%) | 1 (0.7%) |  |  |  |
| **Lifetime LSD use** *(%)* |  |  |  |  |  |
| 0 | 163 (99.4%) | 154 (100.0%) |  |  |  |
| 1-2 | 1 (0.6%) | - |  |  |  |
| **Lifetime ecstasy (MDMA) use** *(%)* |  |  |  |  |  |
| 0 | 163 (99.4%) | 154 (100.0%) |  |  |  |
| 1-2 | 1 (0.6%) | - |  |  |  |
| **Lifetime narcotics use** *(%)* |  |  |  |  |  |
| 0 | 163 (99.4%) | 154 (100.0%) |  |  |  |
| 1-2 | 1 (0.6%) | - |  |  |  |
| **Lifetime tranquillisers or sedatives use** *(%)* |  |  |  |  |  |
| 0 | 162 (98.8%) | 153 (99.35%) |  |  |  |
| 1-2 | 2 (1.2%) | 1 (0.7%) |  |  |  |
|  | | | | | |

**Table S3.**

*Results of the logistic regressions assessing baseline (14 years) predictors of future cannabis use at 19 years old*

|  |  | **Block 1** | | | **Block 2** | | | **Block 3** | | |
| --- | --- | --- | --- | --- | --- | --- | --- | --- | --- | --- |
| Model | Predictors | β | SE | p | β | SE | p | β | SE | p |
| **Psychopathology** |  |  | | |  |  |  |  |  |  |
| Externalizing | Conduct problems | 1.30 | 0.08 | .002 | 1.23 | 0.09 | .002 | 1.35 | 0.09 | .001 |
|  | Hyperactivity | 0.97 | 0.06 | .631 | 0.97 | 0.06 | .623 | 0.96 | 0.06 | .529 |
| Internalizing | Emotional Symptoms | - | - | - | 1.01 | 0.06 | .126 | 1.10 | 0.06 | .126 |
| Others | Peer problems | - | - | - | - | - | - | 0.75 | 0.08 | <.001 |
|  | Prosocial behavior | - | - | - | - | - | - | 0.93 | 0.07 | .341 |
| **Cognitive functions** |  |  |  |  |  |  |  |  |  |  |
|  | Block Design | 0.71 | 0.15 | .026 | - | - | - | - | - | - |
|  | Digit Span | 1.02 | 0.16 | .888 | - | - | - | - | - | - |
|  | Matrix Reasoning | 1.09 | 0.15 | .568 | - | - | - | - | - | - |
|  | Similarities | 1.24 | 0.18 | .244 | - | - | - | - | - | - |
|  | Vocabulary | 1.17 | 0.18 | .383 | - | - | - | - | - | - |
| **Brain activity** |  |  |  |  |  |  |  |  |  |  |
| Anticipation (Nac) | Anticipation hit | 0.92 | 0.14 | .558 | - | - | - | - | - | - |
|  | Anticipation missed | 1.11 | 0.15 | .471 | - | - | - | - | - | - |
| Feedback (PFC) | Feedback hit | 0.72 | 0.40 | .413 | - | - | - | - | - | - |
|  | Feedback missed | 1.78 | 0.38 | .128 | - | - | - | - | - | - |

*Further exploratory analyses*

Upon request from the reviewers, we performed exploratory analyses to compare baseline brain activity of future daily (or near-daily; i.e., ≥ 20 uses in the previous month) cannabis users with their matched controls. From the persistent cannabis users at 22 years old, we were only able to extract beta-weights for reward anticipation (NAc) and feedback (PFC) of 9 and 8 heavy users, respectively. Independent samples t-test revealed no statistically significant group differences (see Table S4).

Additionally, we performed the same analyses with participants that would engage in daily or near-daily cannabis use at age 19. We were then able to extract beta-weights for reward anticipation and feedback of 24 and 22 heavy users, respectively. No statistically significant group differences emerged (see Table S4).

**Table S4.**

*Exploratory independent samples t-tests comparing baseline NAc and PFC activity in future heavy cannabis users and their matched controls*

|  | Matched Controls | Heavy Cannabis users at 22 | Group comparisons | | | Matched Controls | Heavy Cannabis users at 19 | Group Comparisons | | |
| --- | --- | --- | --- | --- | --- | --- | --- | --- | --- | --- |
|  | M (SD) | M (SD) | *t*_(df)_ | *p* | *d* | M (SD) | M (SD) | *t*_(df)_ | *p* | *d* |
| **NAc** |  |  |  |  |  |  |  |  |  |  |
| Gain Anticipation (hit) | 0.68 (1.01) | 1.23 (1.05) | -1.23_(19)_ | .234 | .54 | 0.76 (1.10) | 1.25 (0.96) | -1.58_(43)_ | .122 | .47 |
| Gain Anticipation (missed) | 0.91 (0.96) | 1.28 (0.99) | -0.87_(19)_ | .396 | .38 | 0.66 (1.08) | 0.94 (0.87) | -0.97_(46)_ | .337 | .28 |
| **PFC** |  |  |  |  |  |  |  |  |  |  |
| Hit Feedback | -0.02 (0.44) | 0.17 (0.41) | -0.90_(15)_ | .384 | .44 | 0.06 (0.42) | 0.10 (0.34) | -0.34_(38)_ | .735 | .11 |
| Missed Feedback | -0.17 (0.51) | -0.12 (0.16) | -0.25_(14)_ | .808 | .12 | -0.09 (0.40) | 0.00 (0.27) | -0.85_(39)_ | .400 | .27 |

**Comparing cannabis users and non-users (Q2)**

The descriptive statistics of the sociodemographic variables and the neuropsychological variables, as well as the results of the Chi-square and T-tests comparing the groups on these measures are shown in Table S5. The ANOVA’s results are summarized in Table S6.

For all the ANOVAs, if sphericity was violated (as indicated by the Mauchly’s test), Huynh-Feldt and Greenhouse-Geisser corrected results were reported if Greenhouse-Geisser estimate of sphericity (ε) was >.75 or <.75, respectively. The threshold for statistical significance was set at α = .014.

| **Table S5.**  *Sociodemographic and neuropsychological descriptive statistics (and group comparisons) of persistent cannabis users and matched controls at all timepoints* | | | | | |
| --- | --- | --- | --- | --- | --- |
|  | **Persistent cannabis users**  (*n* = 57) | **Controls**  (*n* = 52) | **Group Comparisons** | | |
|  |  |  | *t*_(df)_ / *X^2^* | *p* | *d/ V* |
| **Age ⁑** *M* (*SD*) | 14.29 (0.39) | 14.34 (0.42) | .618_(107)_ | .538 | .12 |
| [Min., Max.] | [13.00, 15.14] | [13.70, 15.35] |  |  |  |
| **Sex** *n* *(%)* |  |  | 0.072_(1)_ | .788 | .03 |
| Male | 44 (77.2%) | 39 (75.0%) |  |  |  |
| Female | 13 (22.8%) | 13 (25.0%) |  |  |  |
| **Pubertal Development ⁑** *M (SD)* |  |  |  |  |  |
| Male | 2.69 (0.46) | 2.57 (0.49) | -1.089_(80)_ | .279 | .24 |
| Female | 4.05 (0.27) | 4.19 (0.23) | 1.483_(24)_ | .151 | .58 |
| **Language** (%) |  |  | 2.596_(2)_ | .273 | .15 |
| French | 15 (26.3%) | 11 (21.2%) |  |  |  |
| English | 20 (35.1%) | 13 (25.0%) |  |  |  |
| German | 22 (38.6%) | 28 (53.8%) |  |  |  |
| **Socioeconomic status ⁑** *M (SD)* | 2.82 (2.49) | 2.85 (2.84) | 0.048_(106)_ | .962 | .01 |
|  |  |  |  |  |  |
| BL (14 years old) |  |  |  |  |  |
| **SDQ (self-report)** *M (SD)* |  |  |  |  |  |
| Total difficulties | 10.14 (4.81) | 8.81 (4.40) | -1.505_(107)_ | .135 | .30 |
| Emotional Symptoms | 2.51 (2.30) | 2.25 (1.91) | -0.636_(107)_ | .526 | .12 |
| Conduct Problems | 2.37 (1.43) | 1.48 (1.11) | -3.586_(107)_ | <.001 | .69 |
| Hyperactivity/Inattention | 3.53 (1.86) | 2.83 (2.15) | -1.820_(107)_ | .072 | .35 |
| Peer Problems | 1.74 (1.48) | 2.25 (1.62) | 1.727_(107)_ | .087 | .33 |
| Prosocial | 7.28 (1.96) | 7.87 (1.56) | 1.711_(107)_ | .090 | .33 |
| **WISC-IV*** *M (SD)* |  |  |  |  |  |
| Block Design | -0.01 (1.08) | 0.27 (1.01) | 1.369_(102)_ | .174 | .27 |
| Digit Span | 0.12 (0.93) | -0.17 (1.07) | -1.472_(105)_ | .144 | .29 |
| Matrix Reasoning | 0.04 (1.17) | 0.18 (0.95) | 0.633_(102)_ | .528 | .13 |
| Similarities | 0.21 (0.88) | 0.24 (0.93) | 0.146_(103)_ | .884 | .03 |
| Vocabulary | 0.11 (0.83) | 0.22 (0.86) | 0.639_(102)_ | .524 | .13 |
| **Mean FD** *M (SD)* | 0.21 (0.92) | 0.24 (0.11) | 1.207_(62)_ | .232 | .10 |
|  |  |  |  |  |  |
| FU1 (19 years old) |  |  |  |  |  |
| **SDQ (self-report)** *M (SD)* |  |  |  |  |  |
| Total difficulties | 10.77 (5.23) | 8.90 (4.23) | -2.037_(107)_ | .044 | .39 |
| Emotional Symptoms | 2.93 (2.50) | 2.69 (1.99) | -0.546_(107)_ | .586 | .11 |
| Conduct Problems | 2.05 (1.32) | 1.46 (1.04) | -2.588_(107)_ | .011 | .50 |
| Hyperactivity/Inattention | 3.91 (1.87) | 2.88 (2.03) | -2.752_(107)_ | .007 | .53 |
| Peer Problems | 1.88 (1.46) | 1.87 (1.28) | -0.045_(107)_ | .965 | .01 |
| Prosocial | 8.21 (1.62) | 8.37 (1.48) | 0.519_(107)_ | .605 | .10 |
| **Mean FD** *M (SD)* | 0.14 (0.06) | 0.16 (0.06) | 1.125_(62)_ | .265 | .01 |
|  |  |  |  |  |  |
| FU2 (22 years old) |  |  |  |  |  |
| **SDQ (self-report)** *M (SD)* |  |  |  |  |  |
| Total difficulties | 9.54 (4.96) | 7.42 (4.11) | -2.399_(106)_ | .018 | .46 |
| Emotional Symptoms | 2.68 (2.41) | 1.96 (1.74) | -1.760_(106)_ | .081 | .34 |
| Conduct Problems | 1.71 (1.20) | 1.27 (1.03) | -2.058_(106)_ | .042 | .40 |
| Hyperactivity/Inattention | 3.25 (2.08) | 2.37 (1.83) | -2.340_(106)_ | .021 | .45 |
| Peer Problems | 1.89 (1.33) | 1.83 (1.42) | -0.249_(106)_ | .804 | .05 |
| Prosocial | 8.27 (1.52) | 8.52 (1.66) | 0.821_(106)_ | .414 | .16 |
| **WAIS-IV*** *M (SD)* |  |  |  |  |  |
| Block Design | 0.13 (1.23) | 0.40 (0.69) | 1.174_(72)_ | .244 | .27 |
| Digit Span | 0.14 (0.97) | 0.00 (1.50) | -0.183_(11)_ | .858 | .11 |
| Matrix Reasoning | -0.01 (1.18) | 0.41 (0.90) | 1.735_(72)_ | .087 | .40 |
| Similarities | 0.27 (0.88) | 0.22 (0.90) | -0.244_(72)_ | .808 | .06 |
| Vocabulary | 0.02 (1.09) | 0.23 (0.99) | 0.872_(71)_ | .386 | .20 |
| **Mean FD** *M (SD)* | 0.13 (0.05) | 0.14 (0.05) | 0.888_(62)_ | .378 | .05 |
|  |  |  |  |  |  |
| *Note: SDQ = strengths and difficulties questionnaire; WISC = Wechsler Intelligence Scale for Children – Fourth Edition (WISC-IV), FD = Framewise Displacement. Only 8 controls and 5 cannabis users completed the Digit Span subtest at both timepoints* ⁑ *Measured at baseline.*  **Z-scores.* | | | | | |

*Missing Data*

One participant from the cannabis users’ group did not have SDQ data. Twenty controls did not have WISC/WAIS data of the Block Design, Matrix, Reasoning, and Similarities subtests, and 20, 21, and 19 cannabis users did not have the Block Design and Matrix Reasoning subtests, the Vocabulary, and Similarities subtests, respectively. Only 8 controls and 5 cannabis users had completed the Digit Span subtest at both timepoints, as such, the ANOVA was not performed on this measure. Regarding fMRI data, 35 cannabis users and 34 controls had imaging data at all timepoints. One control at FU1, and 4 controls at FU2 had excessive head movement and were excluded from the analyses. One control was excluded for not having data on a contrast of interest.

*Substance Use Descriptive Statistics*

Descriptive statistics regarding substance use patterns at FU1 (19 years) and FU2 (22 years) are shown in Supplementary Table S7. At 19 years old, 22.8% of cannabis users (*n* = 13) reported having used cannabis daily or near daily (≥ 20 times) in the previous month; and at 22 years old, this number increased to 28.1% (*n* = 16). At 19 years old, 36.8% of cannabis users (*n* = 21) reported that cannabis was the only illicit substance they used, and at 22 years old, this number decreased to 24.6% (*n* = 14).

**Table S6.**

*Results of the ANOVAs comparing persistent cannabis users and their matched controls*

| **Measures** | **Main Effect** | **df** | **F** | **p** | **η²** | **Observed Power** | **Pairwise Comparisons** |
| --- | --- | --- | --- | --- | --- | --- | --- |
| **Psychopathology (SDQ)** |  |  |  |  |  |  |  |
| Total Difficulties | Time | 1.81, 191.79 | 5.17 | .008 | 0.05 | 0.79 |  |
|  | Time * Group | 1.81, 191.79 | 0.58 | .542 | 0.01 | 0.14 | **BL, FU1, FU2:** CAN = CON |
|  | Between Subjects | 1, 106 | 5.51 | .021 | 0.05 | 0.64 |  |
| Emotional Symptoms | Time | 1.90, 201.06 | 3.71 | .028 | 0.03 | 0.66 |  |
|  | Time * Group | 1.90, 201.06 | 0.99 | .371 | 0.01 | 0.22 | **BL, FU1, FU2:** CAN = CON |
|  | Between Subjects | 1, 106 | 1.27 | .263 | 0.01 | 0.20 |  |
| Conduct Problems | Time | 1.92, 203.17 | 6.07 | .003 | 0.05 | 0.87 |  |
|  | Time * Group | 1.92, 203.17 | 1.73 | .182 | 0.02 | 0.35 | **BL, FU1**: CAN > CON; **FU2:** CAN=CON |
|  | Between Subjects | 1, 106 | 13.27 | <.001 | 0.11 | 0.95 |  |
| Hyperactivity/Inattention | Time | 1.90, 201.22 | 3.70 | .029 | 0.03 | 0.66 |  |
|  | Time * Group | 1.90, 201.22 | 0.37 | .681 | 0.00 | 0.11 | **FU1**: CAN > CON |
|  | Between Subjects | 1, 106 | 9.04 | .003 | 0.08 | 0.85 |  |
| Peer Problems | Time | 1.94, 205.41 | 0.42 | .649 | 0.00 | 0.12 |  |
|  | Time * Group | 1.94, 205.41 | 2.78 | .066 | 0.26 | 0.54 | **BL, FU1, FU2:** CAN = CON |
|  | Between Subjects | 1, 106 | 0.44 | .510 | 0.00 | 0.10 |  |
| Prosocial Score | Time | 2, 212 | 14.44 | <.001 | 0.12 | 1.00 |  |
|  | Time * Group | 2, 212 | 0.95 | .390 | 0.01 | 0.21 | **BL, FU1, FU2:** CAN = CON |
|  | Between Subjects | 1, 106 | 9.98 | .164 | 0.02 | 0.29 |  |
| **Cognition** |  |  |  |  |  |  |  |
| Block Design | Time | 1, 67 | 0.56 | .459 | 0.01 | 0.11 |  |
|  | Time * Group | 1, 67 | 0.01 | .894 | 0.00 | 0.05 | **BL, FU2:** CAN = CON |
|  | Between Subjects | 1, 67 | 1.25 | .268 | 0.02 | 0.20 |  |
| Matrix Reasoning | Time | 1, 67 | 0.95 | .334 | 0.01 | 0.16 |  |
|  | Time * Group | 1, 67 | 0.51 | .477 | 0.01 | 0.11 | **BL, FU2:** CAN = CON |
|  | Between Subjects | 1, 67 | 3.11 | .082 | 0.04 | 0.41 |  |
| Similarities | Time | 1, 68 | 0.01 | .927 | 0.00 | 0.05 |  |
|  | Time * Group | 1, 68 | 0.01 | .944 | 0.00 | 0.05 | **BL, FU2:** CAN = CON |
|  | Between Subjects | 1, 68 | 0.15 | .699 | 0.00 | 0.07 |  |
| Vocabulary | Time | 1, 66 | 0.17 | .683 | 0.00 | 0.07 |  |
|  | Time * Group | 1, 66 | 0.24 | .628 | 0.00 | 0.08 | **BL, FU2:** CAN = CON |
|  | Between Subjects | 1, 66 | 0.69 | .409 | 0.01 | 0.13 |  |

**Recovery with at least one month of abstinence (Q.2.1)**

*Psychopathology*

**Conduct problems**. There was no main effect of time, *F*(2, 100) = 1.73, *p* = .183, *η^2^* = 0.03, no significant time*group interaction, *F*(4, 100) = 0.92, *p* = .457, *η^2^* = 0.04, and no between-subjects effect, *F*(1, 50) = 4.11, *p* = .022, *η^2^* = 0.14. Pairwise comparisons revealed that cannabis users had significantly higher conduct problems’ scores (*M* = 1.06, *SD* = 0.25) compared to controls (*M* = 0.94, *SD* = 0.25) at 22 years old (*p* = .003).

**Hyperactivity/inattention**. There was no main effect of time, *F*(2, 100) = 1.79, *p* = .409, *η^2^* = 0.02, no significant time*group interaction, *F*(4, 100) = 0.86, *p* = .491, *η^2^* = 0.03, and no significant between-subjects effect, *F*(1, 50) = 1.44, *p* = .246, *η^2^* = 0.06.

*Substance Use Descriptive Statistics*

At 22 years old, 9 abstinent cannabis users (47.4%) had not used cannabis over a year, 7 (36.8%) reported having used 1-2 times, 1 (5.3%) used 3-5 times, 1 (5.3%) used 6-9 times, and 1 (5.3%) 10-19 times, but none had used in the previous month.

At 19 years old (when the abstinent users were still using cannabis), they exhibited a smaller amount of cannabis use than those who remained cannabis users at 22 years old (a subsample of 17 users). Whereas 23.6% of future persistent cannabis users reported using cannabis daily or near daily at 19 years, only 5.3% of future abstinent users did. In the 12 months prior to the FU1 assessment, 52.9% of future persistent cannabis users versus 10.5% of future abstinent users reported having used cannabis more than 40 times.

**Table S8.**

*Descriptive statistics of the sociodemographic and neuropsychological variables of each group (persistent cannabis users, abstinent cannabis users, non-users).*

|  | **Persistent Cannabis users**  (*n* = 17) | **Abstinent Cannabis Users** | **Controls**  (*n* = 17) |
| --- | --- | --- | --- |
|  |  | (n = 19) |  |
| **Age ⁑** *M* (*SD*) | 14.37 (0.44) | 14.34 (0.48) | 14.31 (0.40) |
| [Min., Max.] | [13.71, 15.14] | [13.53, 15.13] | [13.87, 14.92] |
| **Sex** *n* *(%)* |  |  |  |
| Male | 13 (76.47%) | 12 (63.16%) | 10 (58.82%) |
| Female | 4 (23.53%) | 7 (36.84%) | 7 (41.18%) |
| **Pubertal Development ⁑** *M (SD)* |  |  |  |
| Male | 2.58 (0.41) | 2.63 (0.54) | 2.78 (0.37) |
| Female | 4.00 (0.31) | 4.05 (0.22) | 4.14 (0.20) |
| **Language** (%) |  |  |  |
| French | 8 (47.06%) | 6 (31.58%) | 2 (11.76%) |
| English | 0 (0.00%) | 1 (5.26%) | 5 (29.41%) |
| German | 9 (52.94%) | 12 (63.16%) | 10 (58.82%) |
| **Socioeconomic status ⁑** *M (SD)* | 2.59 (2.60) | 2.47 (2.06) | 2.18 (2.07) |
|  |  |  |  |
| BL (14 years old) |  |  |  |
|  |  |  |  |
| **SDQ (self-report)** *M (SD)* |  |  |  |
| Total difficulties | 8.76 (4.42) | 7.95 (3.44) | 8.59 (3.30) |
| Emotional Symptoms | 1.76 (2.11) | 2.05 (1.39) | 2.53 (2.27) |
| Conduct Problems | 2.18 (1.47) | 2.00 (1.67) | 1.18 (0.64) |
| Hyperactivity/ Inattention | 3.29 (1.61) | 3.05 (1.84) | 3.18 (2.16) |
| Peer Problems | 1.53 (1.33) | 0.84 (1.12) | 1.71 (1.31) |
| Prosocial | 7.53 (2.10) | 7.63 (1.46) | 7.88 (1.90) |
| **WISC-IV*** *M (SD)* |  |  |  |
| Block Design | -0.14 (1.17) | 0.41 (0.76) | 0.24 (0.85) |
| Digit Span | 0.13 (0.76) | 0.50 (0.72) | -0.17 (1.00) |
| Matrix Reasoning | 0.04 (0.97) | 0.31 (0.72) | 0.13 (0.93) |
| Similarities | 0.07 (0.79) | 0.73 (0.44) | 0.15 (1.06) |
| Vocabulary | -0.06 (1.18) | 0.81 (0.64) | 0.45 (0.79) |
|  |  |  |  |
| FU1 (19 years old) |  |  |  |
|  |  |  |  |
| **SDQ (self-report)** *M (SD)* |  |  |  |
| Total difficulties | 10.18 (4.99) | 7.95 (4.55) | 8.00 (3.66) |
| Emotional Symptoms | 2.65 (2.60) | 2.42 (1.89) | 2.24 (1.86) |
| Conduct Problems | 1.76 (1.20) | 1.42 (1.39) | 1.29 (0.85) |
| Hyperactivity/ Inattention | 3.82 (1.91) | 2.68 (1.92) | 2.59 (1.70) |
| Peer Problems | 1.94 (1.48) | 1.42 (1.30) | 1.88 (1.27) |
| Prosocial | 8.65 (1.22) | 8.37 (1.54) | 8.65 (1.32) |
|  |  |  |  |
| FU2 (22 years old) |  |  |  |
|  |  |  |  |
| **SDQ (self-report)** *M (SD)* |  |  |  |
| Total difficulties | 10.35 (5.13) | 7.32 (4.19) | 7.59 (4.85) |
| Emotional Symptoms | 2.76 (2.49) | 1.84 (1.92) | 2.12 (1.93) |
| Conduct Problems | 2.06 (1.30) | 1.37 (0.96) | 0.94 (0.83) |
| Hyperactivity/ Inattention | 3.41 (2.32) | 2.37 (1.86) | 2.65 (1.84) |
| Peer Problems | 2.12 (1.58) | 1.74 (2.18) | 1.88 (1.83) |
| Prosocial | 8.53 (1.42) | 8.42 (1.35) | 8.65 (1.46) |
| **WAIS-IV*** *M (SD)* |  |  |  |
| Block Design | -0.42 (1.70) | 0.07 (0.88) | 0.39 (0.72) |
| Digit Span | 0.70 (0.00) | 0.63 (1.35) | -0.29 (1.40) |
| Matrix Reasoning | -0.20 (2.02) | 0.51 (1.00) | 0.37 (1.04) |
| Similarities | 0.11 (0.86) | 0.62 (0.66) | 0.48 (0.77) |
| Vocabulary | -0.25 (1.44) | 0.57 (0.93) | 0.61 (0.66) |
|  |  |  |  |

**Z-scores.*
